# Massive Pulmonary Thromboembolism and Stroke

**DOI:** 10.1155/2011/398571

**Published:** 2011-09-28

**Authors:** Poobalan Naidoo, Richard Hift

**Affiliations:** Department of Medicine, Nelson R Mandela School of Medicine, UKZN, 4013 KwaZulu-Natal, South Africa

## Abstract

A 38-year-old HIV-positive female, recently started on antiretroviral therapy, presented in extremis. She had features suggestive of an HIV-associated cardiomyopathy complicated by the following problems: a four-day-old stroke, extensive deep venous thrombosis, and massive pulmonary embolism. She received intravenous streptokinase with rapid improvement, both haemodynamically and, unexpectedly, neurologically. Our case illustrates that a positive outcome is potentially possible where the two conditions coincide.

## 1. Case Report

A 38-year-old female presented with a 2-day history of dyspnoea and pleuritic chest pain. She had developed a right sided hemiplegia 4 days before the current presentation. She was HIV positive with a CD4 count of 180, and she was receiving highly active antiretroviral therapy.

On examination she was distressed, with a respiratory rate of 30 breaths per minute, a low-volume pulse of 130 beats per minute, and a blood pressure of 70/30 mm Hg. The jugular venous pressure was markedly elevated. On palpation, the apex beat was myopathic and displaced, there was a left parasternal heave and palpable pulmonary component of the second heart sound. Auscultation revealed a loud pulmonary component of the second heart sound (P2) and a third heart sound at the left sternal edge. Her right leg was markedly swollen. She had a dense right hemiplegia with absent power on the right. 

Chest X-ray showed an increased cardiothoracic ratio. ECG showed a sinus tachycardia and left bundle branch block without features of acute right ventricular strain. Doppler ultrasound confirmed an extensive right-sided deep vein thrombosis. 

**Figure 1:**
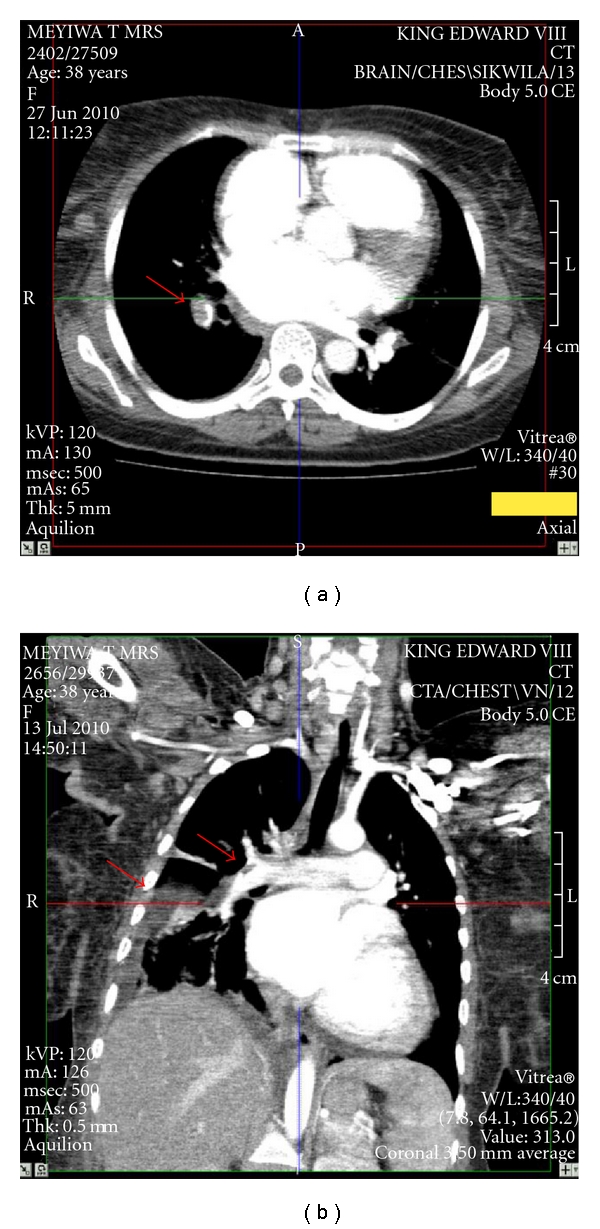
(a) Axial computerised tomography pulmonary angiogram showing filling defect in pulmonary artery. Similar filling defects were found at multiple levels of the pulmonary artery vasculature. (b) Coronal computerised tomography pulmonary angiogram showing multiple filling defects in the pulmonary artery and a triangular hypodensity in the right lower lung, which is most likely a pulmonary infarct.

**Figure 2:**
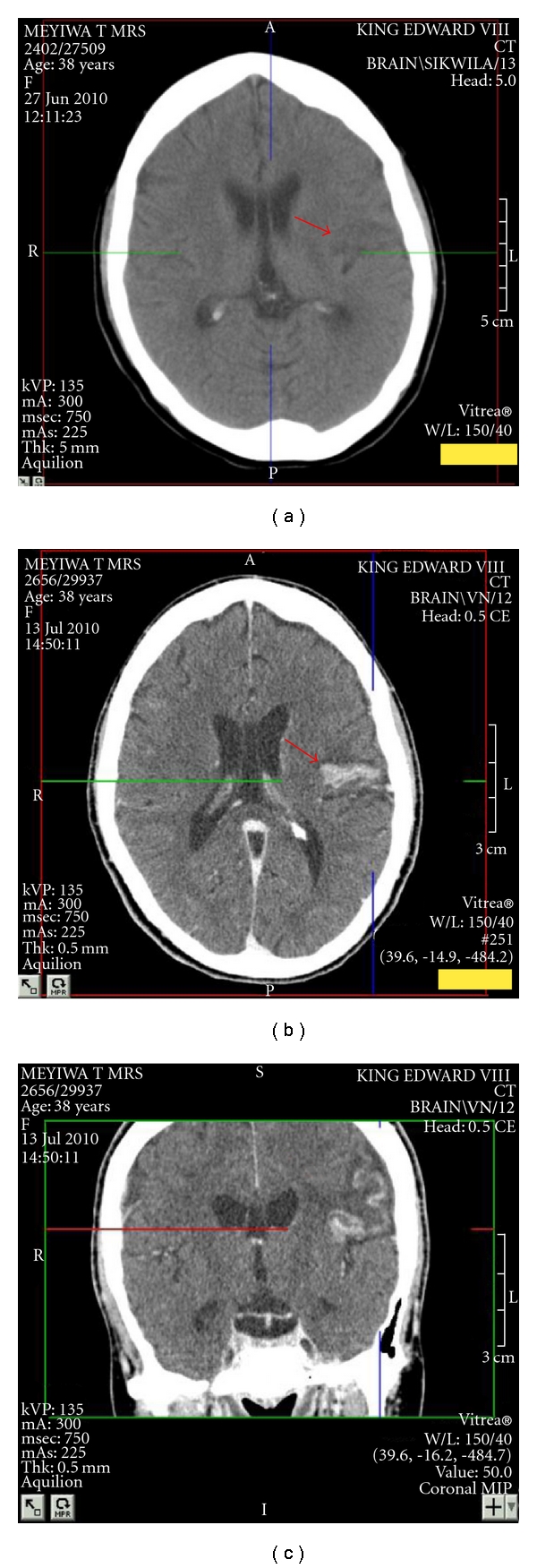
(a) Axial computerised tomography (unenhanced) brain showing a wedge-shaped hypodensity in the left frontopareital area with extension across both gray and white matter, which is in keeping with a cerebral infarct. (b) Axial computerised tomography (enhanced) brain showing hyperdensity in the left-hand side in the area of previous ischaemia. (c) Coronal computerised tomography (enhanced) brain showing hyperdensity in the left-hand side in the area of previous ischaemia.

A helical CT pulmonary angiogram demonstrated multiple defects in major branches of both pulmonary arteries and multiple areas of pulmonary infarction (Figure 1). A CT brain scan demonstrated recent left frontoparietal cerebral infarction (Figure 2). Echocardiography showed four-chamber dilatation, a left ventricular ejection fraction of 13%, mitral regurgitation, and tricuspid regurgitation. Pulmonary artery systolic pressure was 67 mm Hg. There was no evidence of intramural thrombosis and no direct communication between the right and left side of the heart was demonstrated. 

Full blood count and routine chemistry were unremarkable. A D-dimer test was positive and troponin T concentration was normal. Our final diagnosis was that of HIV infection, probable HIV-associated cardiomyopathy, deep venous thrombosis, and acute massive pulmonary thromboembolism. 

The patient was unstable, tachycardic, and severely hypotensive. Though mindful of her stroke, we administered streptokinase according to the standard protocol. Her haemodynamic status improved markedly, and blood pressure normalised. Signs of pulmonary hypertension improved. A repeat CT pulmonary angiogram showed partial resolution of the pulmonary emboli. To our surprise, her neurological signs improved remarkably as well, with an improvement in muscle power over several hours to near normal. A repeat CT brain scan now demonstrated a rim of enhancement around the infarction, reported as a haemorrhage, though subsequently suggested to represent an area of luxury perfusion. 

She maintained her initial clinical improvement and improved further over subsequent days on standard therapy for heart failure. Intercostal drainage was required for a large right haemorrhagic pleural effusion secondary to pulmonary infarction. She was discharged after 18 days on warfarin. 

## 2. Discussion

Risk factors for thrombosis in our patient included immobility, dilated cardiomyopathy, and her HIV status. HIV is an independent risk factor for thrombosis. Antiretroviral therapy does not appear to significantly increase risk of venous thromboembolism in HIV-positive patients [1]. The prothrombotic state in HIV-positive patients correlates with the severity of HIV-associated immunosuppression. The mechanism of this increased prothrombotic state in HIV patients is unknown. However, various abnormalities predisposing to a prothrombotic state have been reported, including the presence of antiphospholipid antibodies and lupus anticoagulant, deficiencies of protein C, protein S, heparin cofactor II, and antithrombin, and increased levels of von Willebrand factor and D-dimers [2, 3]. 

Our patient demonstrated global myocardial impairment, probably on the basis of an HIV-associated cardiomyopathy. Her sudden deterioration and acute onset of pulmonary hypertension suggested the onset of “clinically massive” or high-risk pulmonary embolism [4], which is associated with a high short-term mortality [4], especially during the first few hours [5]. There is consensus that such patients should be offered urgent recanalization of the pulmonary arteries, whether by thrombolysis or by mechanical means, in order to reverse right ventricular pressure overload and failure [5, 6]. 

An ischaemic stroke within the past 6 months is accepted as an absolute contraindication to thrombolysis [4], though consensus guidelines note that even absolute contraindications may be considered relative in patient with immediately life-threatening high-risk pulmonary embolism. 

We believed our patient to be unsuitable for embolectomy. With her informed consent, we proceeded to thrombolysis despite the stroke. In response to thrombolysis, the patient improved dramatically and repeat CT angiography revealed partial resolution of the thrombi. To our surprise, there was a prompt, marked improvement in the patient's neurological status. 

Thrombolysis is now recommended as initial treatment for ischaemic stroke presenting within 3 hours [7] or 4.5 hours [8]. Therapy carries a risk of symptomatic intracranial haemorrhage less than 7% [7]. Currently no authority recommends thrombolysis four days after the onset of stroke. 

There are three case reports of patients with coexistent pulmonary embolism and stroke given thrombolysis. Pelletier et al. [9], Pavesi et al. [10], and Allport and Butcher [11] reported patients with pulmonary embolus and paradoxical embolism resulting in acute stroke, of recent onset, who improved (neurologically and haemodynamically) following thrombolysis. All patients received thrombolysis within hours of first presentation. Unfortunately, we did not perform provocation tests in our patient, and thus we were not able to rule out a transient right-to-left shunt. Perhaps, similar to the above-mentioned cases, our patient also had a paradoxical embolism. Our patient differs from aforementioned case reports in that she only received thrombolysis 4 days following the onset of neurological symptoms. Thrombolysis appeared to result in significant improvement in both the pulmonary embolism and the stroke. Though the neurological improvement may be coincidental, we believe that thrombolysis may have allowed reperfusion of a penumbra of critical cerebral ischaemia, possibly augmented by an increase in cerebral perfusion pressure as her haemodynamic status improved. 

During the evolution of an ischaemic stroke, there is a gradient of hypoperfusion [12]. Areas with the least perfusion progress to irreversible damage and are termed the “necrotic core.” The remaining hypoperfused tissue is divided into two compartments, that is, the “penumbra and oligaemia.” Tissue within the penumbra is potentially salvageable [13]. Most patients exhibit substantial volumes of penumbra for many hours [14] or exceptionally, days after stroke onset [15]. The oligaemic compartment, suffers a milder degree of hypoperfusion and is not normally at risk of infarction [13]. However, secondary events such as systemic hypotension, intracranial hypertension, or hyperglycaemia may result in the oligaemic area transforming into a penumbral state and even worse, the necrotic core. 

European Cooperative Acute Stroke Study (ECASS) III, a randomized, placebo-controlled, phase-three trial, tested the efficacy and safety of rt-PA administered 3 to 4.5 hours after symptom onset [16]. ECASS III confirmed the National Institute of Neurological Disorders and Stroke (NINDS) [17] study finding of a significant benefit with rt-PA in the primary end point at 3 months. Additionally, rt-PA administered 3–4.5 hours after the onset of stroke symptoms resulted in a significant improvement of clinical outcome without increasing the risk of intracebral haemorrhage. The results of the ECASS III trial and pooled analysis data prompted the American Heart Association/American Stroke Association to advise that rt-PA should be offered between 3–4.5 hours to select patients [18]. The thrombolytic treatment window period for acute stroke is in evolution [19]. It is postulated that physiologic imaging will more effectively guide the selection of patients for thrombolysis and may, for select patients, enable the window period for thrombolysis to increase even beyond 4.5 hours [13]. We postulate that our patient had “characteristics” that allowed for successful thrombolysis so late after initial neurological symptoms. The aforementioned “characteristics” are currently unknown, but advancing knowledge in medical science will perhaps provide answers in the not too distant future. 

In summary, thrombolysis proved safe and effective, and it was accompanied by unexpected neurological improvement. 

## References

[1] Crum-Cianflone NF, Weekes J, Bavaro M (2008). Review: thromboses among HIV-infected patients during the highly active antiretroviral therapy era. *AIDS Patient Care and STDs*.

[2] Wasif Saif H, Greenberg B (2001). HIV and thrombosis: a review. *AIDS Patient Care and STDs*.

[3] Shen YM, Frenkel EP (2004). Thrombosis and a hypercoaguble state in HIV-infected patients. *Clinical and Applied Thrombosis/Hemostasis*.

[4] Torbicki A, Perrier A (2008). Guidelines on the diagnosis and management of acute pulmonary embolism: the Task Force for the Diagnosis and Management of Acute Pulmonary Embolism of the European Society of Cardiology (ESC). *European Heart Journal*.

[5] Lankeit M, Konstantinides S (2010). Mortality risk assessment and the role of thrombolysis in pulmonary embolism. *Clinics in Chest Medicine*.

[6] Todd JL, Tapson VF (2009). Thrombolytic therapy for acute pulmonary embolism: a critical appraisal. *Chest*.

[7] Albers GW, Amarenco P, Easton JD, Sacco RL, Teal P (2008). Antithrombotic and thrombolytic therapy for ischemic stroke: American College of Chest Physicians evidence-based clinical practice guidelines (8th edition). *Chest*.

[8] Marsh JD, Keyrouz SG (2010). Stroke prevention and treatment. *Journal of the American College of Cardiology*.

[9] Pelletier M, Bugeaud R, Ibrahim R, Morency G, Kouz S (2010). Successful thrombolysis of a stroke with a pulmonary embolism in a young woman. *Journal of Emergency Medicine*.

[10] Pavesi PC, Pedone C, Crisci M, Piacentini A, Fulvi M, Di Pasquale G (2008). Concomitant submassive pulmonary embolism and paradoxical embolic stroke after a long flight: which is the optimal treatment?. *Journal of Cardiovascular Medicine*.

[11] Allport LE, Butcher KS (2008). Thrombolysis for concomitant acute stroke and pulmonary embolism. *Journal of Clinical Neuroscience*.

[12] Astrup J, Siesjö BK, Symon L (1981). Thresholds in cerebral ischemia—The ischemic penumbra. *Stroke*.

[13] Moustafa RR, Baron JC (2008). Pathophysiology of ischaemic stroke: insights from imaging, and implications for therapy and drug discovery. *British Journal of Pharmacology*.

[14] Baron JC (1999). Mapping the ischaemic penumbra with PET: implications for acute stroke treatment. *Cerebrovascular Diseases*.

[15] Perez A, Restrepo L, Kleinman JT, Barker P, Beauchamp N, Wityk RJ (2006). Patients with diffusion-perfusion mismatch on magnetic resonance imaging 48 hours or more after stroke symptom onset: clinical and imaging features. *Journal of Neuroimaging*.

[16] Hacke W, Kaste M, Bluhmki E (2008). Thrombolysis with alteplase 3 to 4.5 hours after acute ischemic stroke. *New England Journal of Medicine*.

[17] The National Institute of Neurological Disorders and Stroke rt-PA Stroke Study Group (1995). Tissue plasminogen activator for acute ischemic stroke. *New England Journal of Medicine*.

[18] Del Zoppo GJ, Saver JL, Jauch EC, Adams HP (2009). Expansion of the time window for treatment of acute ischemic stroke with intravenous tissue plasminogen activator: a science advisory from the American heart association/american stroke association. *Stroke*.

[19] Stemer A, Lyden P (2010). Evolution of the thrombolytic treatment window for acute ischemic stroke. *Current Neurology and Neuroscience Reports*.

